# Understanding the effect of vector dynamics in epidemic models using center manifold analysis

**DOI:** 10.1063/1.4756398

**Published:** 2012-09-26

**Authors:** Filipe Rocha, Maíra Aguiar, Max Souza, Nico Stollenwerk

**Affiliations:** Centro de Matemática e Aplicações Fundamentais, Universidade de Lisboa, Portugal; Departamento de Matemática Aplicada, Universidade Federal Fluminense, Niterói, Brazil; Centro de Matemática e Aplicações Fundamentais, Universidade de Lisboa, Portugal; European Society of Computational Methods in Sciences, Engineering, and Technology (ESCMSET), European Academy of Sciences; European Academy of Sciences and Arts European Academy of Arts, Sciences and; European Society of Computational Methods in Sciences, Engineering, and Technology (ESCMSET); Fellow of the IMA; Greece; Technological Educational Institute of Chalkis, Greece; School of Pedagogical & Technological Education (ASPETE), Athens, Greece

## Abstract

In vector borne diseases the human hosts' epidemiology often acts on a much slower time scales than the one of the mosquitos which transmit the disease as a vector from human to human, due to their vastly different life cycles. We investigate in a model with susceptible (S), infected (I) and recovered (R) humans and susceptible (U) and infected (V) mosquitoes in how far the fast time scale of the mosquito epidemiology can be slaved by the slower human epidemiology, so that for the understanding of human disease data mainly the dynamics of the human time scale is essential and only slightly perturbed by the mosquito dynamics. This analysis of the SIRUV model is qualitatively in agreement with a previously investigated simpler SISUV model, hence a feature of vector-borne diseases in general.

## INTRODUCTION

Modelling vector-borne diseases and as one of the main examples dengue fever has attracted the attention of biomathematics researchers since a long time. Dengue fever and dengue hemorrhagic fever are vector-borne diseases caused by an arbovirus with 4 serotypes, which infects millions of people every year in tropical and subtropical areas. The main vector of the disease is the mosquito *Aedes aegypti* which is well adapted to live in human environments. This mosquito species lives in tropical and subtropical areas, but it is spreading its habitat thanks to the global changes and also the globalization of the humans societies. The fast spreading that has been observed in recent years is expanding the risk areas so, in order to avoid new outbreaks, research interest is increasing to prevent disease spreading by e.g. vaccine development and modelling and predicting the spread of disease.

In this paper we investigate the interplay between mosquitos and humans dynamics in vector-born diseases in the SIRUV setting, hence modelling transitions between susceptible (S), infected (I) and recovered (R) humans and susceptible (U) and infected (V) mosquitoes, after breifly reviewing earlier results in a simplified SISUV setting, in which geometric insights are easier obtained than in the SIRUV model.

## THE SIMPLEST MODEL FOR VECTOR-BORNE DISEASES

For the human disease epidimiology we assume in the simplest case an SIS model (susceptibles, infected and again susceptibles) for the human epidemiological system coupled with the dynamic of the vectors, which can be susceptible (U) or infected (V). This gives the SISUV model as simplest coupled human-vector system

$$\frac{d}{dt}S = \alpha I - \frac{\beta}{M}SV \quad , \quad \frac{d}{dt}I = \frac{\beta}{M}SV - \alpha I \quad , \quad \frac{d}{dt}U = \psi - \nu U - \frac{\vartheta}{N}UI \quad , \quad \frac{d}{dt}V = \frac{\vartheta}{N}UI - \nu V \tag{1}$$

where $\alpha$ is the recovery rate, $\beta$ is the infection rate, $\vartheta$ is the infection rate of mosquitos, $\psi$ is the number of mosquitos that are born and $\nu$ is the death rate of mosquitos. If we consider constant population sizes for the humans $N = S(t) + I(t)$ and for the mosquitos $M = U(t) + V(t)$ it is possible to simplify the model to a two dimensional dynamical system, given by

$$\frac{d}{dt}I = \frac{\beta}{M}(N - I)V - \alpha I \quad , \quad \frac{d}{dt}V = \frac{\vartheta}{N}(M - V)I - \nu V \quad . \tag{2}$$

A trajectory of the system is ploted in the figure 1 (red curve) starting from an arbitrary initial condition. In this plot it is possible to verify that the trajectory initially only goes in $V$ direction, before it goes into a curve which possibly

*Numerical Analysis and Applied Mathematics ICNAAM 2012*
AIP Conf. Proc. 1479, 1319-1322 (2012); doi: 10.1063/1.4756398
© 2012 American Institute of Physics 978-0-7354-1091-6/$30.00

represents a fixed relationship between the state space variables as will be analyzed below. A closer look at these parameters shows that the parameters for the mosquito dynamics $\nu = 36.5\mathrm{y}^{-1}$ and $\vartheta = 73\mathrm{y}^{-1}$ are much larger than the parameters for the human dynamics $\alpha = 0.1\mathrm{y}^{-1}$ and $\beta = 0.2\mathrm{y}^{-1}$. To describe this separation of time scales we set $\nu =: \frac{\bar{\nu}}{\varepsilon}$ with $\bar{\nu}$ in the scale of $\alpha$ and $\beta$, e.g. we can set exactly $\bar{\nu} := \alpha$ giving $\varepsilon = \frac{\bar{\nu}}{\nu} = \frac{0.1}{36.5}$. Then we set $\vartheta =: \frac{\bar{\vartheta}}{\varepsilon}$ giving $\bar{\vartheta} = \frac{\vartheta}{\nu}\alpha$. With these parameters we obtain for the ODE system the form

$$\frac{d}{dt}I = \frac{\beta}{M}(N-I)V - \alpha I \quad , \quad \frac{d}{dt}V = \frac{1}{\varepsilon}\left(\frac{\bar{\vartheta}}{N}(M-V)I - \bar{\nu}V\right) \tag{3}$$

with different time scales, slow for the $I$ dynamics and fast for the $V$ dynamics. This system has been analyzed in [1] as slow-fast dynamics, giving as result a relation between the fast variable $V$ as function of the slow variable $I$ as

$$V(I) = \frac{\frac{\bar{\vartheta}}{N}I}{\frac{\bar{\vartheta}}{N}I + \bar{\nu}} \cdot M \quad . \tag{4}$$

In order to understand the general set-up also in systems where a simple separation into fast and slow ODEs is not possible, like it will be the case of the SIRUV-model, we now apply an approximate center manifold analysis around the endemic fixed point, which again gives the information of the manifold in which the slow dynamics finally evolves. In order to obtain the center manifold for the slow dynamics we first analyze the endemic fixed point and the eigenvectors and eigenvalues of the Jacobian matrix around the fixed point given by Eq. (4) and $I^* = (\beta - \alpha \cdot \frac{\nu}{\vartheta})N/(\beta + \alpha)$ giving

$$A = \begin{pmatrix} -\frac{\beta}{M} \cdot V^* - \alpha & \frac{\beta}{M} \cdot (N - I^*) \\ \frac{\bar{\vartheta}}{\varepsilon N} \cdot (M - V^*) & \frac{1}{\varepsilon}\left(-\frac{\bar{\vartheta}}{N} \cdot I^* - \bar{\nu}\right) \end{pmatrix} =: \begin{pmatrix} a & b \\ c & d \end{pmatrix} \quad . \tag{5}$$

From the Jacobian Matrix we calculate the eigenvalues and the eigenvectors for $i = 1, 2$

$$\lambda_{1/2} = \frac{(a+d)}{2} \pm \sqrt{\left(\frac{a+d}{2}\right)^2 - (ad - bc)} \quad , \quad \underline{u_i} = \frac{1}{\sqrt{1 + \left(\frac{c}{d-\lambda_i}\right)^2}} \begin{pmatrix} 1 \\ -\frac{c}{d-\lambda_i} \end{pmatrix} \quad . \tag{6}$$

Inspecting the numerical values it is possible to verify that one eigenvalue is close to 0 ( $\lambda_1 \approx 0$ ) and another one is largely negative ( $\lambda_2 \approx d << 0$ ). This approximation implies that the second eigenvector is $\underline{u_2} \approx \begin{pmatrix} 0 \\ 1 \end{pmatrix}$. So the eigenvector correspondent to $\lambda_1 = 0$ is $\underline{u_1} = \frac{1}{k}\begin{pmatrix} 1 \\ -\frac{c}{d} \end{pmatrix}$ with $k := \sqrt{1 + \left(\frac{c}{d}\right)^2}$. In figure 1 a) we plot the eigenvector directions around the fixed point. It is possible to see that the second eigendirection is almost in $V$ direction and the other one is tangent to the curve $V(I)$.

In order to perform the center manifold analysis the $(I, V)$ coordinate system was shifted into a $(x, y)$ system with the global fixed point at the origin and the eigenvectors as coordinate axis. Thsi transformation in done considering $\underline{x} := T^{-1}\underline{z}$, where the vector $\underline{z}$ is defined by the coordinates $z := I - I^*$ , $w := V - V^*$ and the matrix $T^{-1} = \begin{pmatrix} k & 0 \\ \frac{c}{d} & 1 \end{pmatrix}$, comes from the matrix $T$ of the eigenvectors. With these values it is possible to obtain the ODE of the transformed system

$$\dot{x} = -\frac{\beta}{M}xy + \frac{\beta}{M}\frac{c}{d \cdot k}x^2 \quad , \quad \dot{y} = d \cdot y + \left(\frac{\beta}{M}\frac{c}{d} + \frac{\vartheta}{N}\right)\left(\frac{c}{d \cdot k^2}x^2 - \frac{1}{k}xy\right) \quad . \tag{7}$$

To find the transformation $y = h(x)$ along the center manifold, the functional $\mathcal{N}(h(x))$ has to vanish

$$\mathcal{N}(h(x)) = \frac{dx}{dt} \cdot f(x, h(x)) - (d \cdot h(x) + g(x, h(x))) = 0 \tag{8}$$

with $h$ having vanishing value and vanishing first derivative at the origin. The functional Eq. (8) can be solved e.g. by comparison of coefficients of a polynomial expansion of $h$. The polynomial approximation

$$h(x) := a_2 \cdot x^2 + a_3 \cdot x^3 + a_4 \cdot x^4 + a_5 \cdot x^5 + \mathcal{O}(x^6) \tag{9}$$

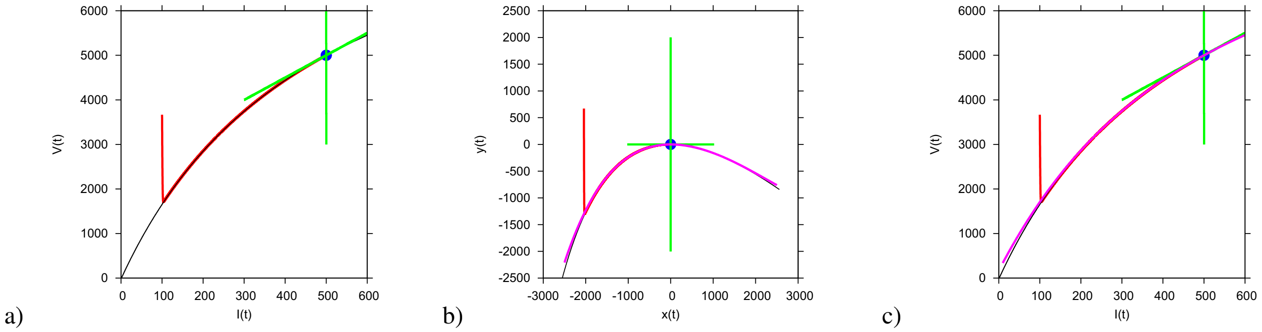

**FIGURE 1.** State space portrait of the SISUV system: The red curve represents the trajectory of the model, the black curve the manifold $V(I)$ and the blue dot is the endemic stationary state. a) shows the eigendirections (green lines). b) shows the transformed system by the center manifold analysis (with the stationary state as the origin and the eigenvectors as the axis). The violet line represents the fifth order polynomial approximation of the center manifold. c) The backtransformation of the center manifold into the original coordinate system $(IV)$

for $y = h(x)$ is given by comparison of coefficients with the results of

$$a_2 = -\frac{c \cdot s}{d^2 \cdot k^2} \quad , \quad a_3 = \frac{1}{d}\left(\frac{2c}{d \cdot k}\frac{\beta}{M} + \frac{s}{k}\right) a_2 \quad , \quad a_j = \frac{1}{d}\left((j-1)\frac{\beta}{M}\frac{c}{k \cdot d} + \frac{s}{k}\right) a_{j-1} - \frac{\beta}{M \cdot d}\left(\sum_{\ell=2}^{j-2} \ell \cdot a_j \cdot a_{j-\ell}\right) \quad (10)$$

for $j = 4, 5, ..., \infty$. The polynomial approximation (shown in the figure 1 b)) fits well to the manifold $V(I)$. From the polynomial approximation it is possible to calculate the backtransformation into the original $(I,V)$ coordinate system (figure 1 c)), thanks to the equation

$$V(I) = V^* - \frac{c}{d} \cdot (I^* - I) + a_2 \left(k \cdot (I - I^*)\right)^2 + a_3 \left(k \cdot (I - I^*)\right)^3 + \mathcal{O}((I - I*)^4) \quad . \quad (11)$$

In a stochastic version of this SISUV system we observe qualitatively the same behaviour as in the deterministic system, namely fast initial trajectory in $V$ direction followed by a slower convergence along the center manifold towards the fixed point [1]. Around the fixed point the stochastic system keeps fluctuating mainly along the center manifold. the stochastic system is formulated in the framework of time continuous Markov processes, in physics often also called master equations [2].

## THE SIRUV MODEL FOR COUPLED HUMAN AND MOSQUITO EPIDEMIOLOGY

The SIRUV model is based on a more complex epidemic model (SIR) which has an additional state (recovered humans). This case was created to consider diseases, such as dengue fever, where humans after recovery become immune to the pathogen. The vectors dynamic remains as before because the mosquitos donot have an immune system, hence cannot recover from the infection. The correspondent ODE system is given by $\frac{d}{dt}S = \mu(N-S) - \frac{\beta}{M}SV$ , $\frac{d}{dt}I = \frac{\beta}{M}SV - (\gamma+\mu)I$ , $\frac{d}{dt}R = \gamma I - \mu R$ , $\frac{d}{dt}U = \psi - \nu U - \frac{\vartheta}{N}UI$ , $\frac{d}{dt}V = \frac{\vartheta}{N}UI - \nu V$ where $\gamma$ and $\mu$ are the birth and death rates of the humans, respectively. For the simulations, these parameters are set as $\gamma = \frac{1}{7\,\mathrm{d}} = \frac{365}{7}\mathrm{y}^{-1}$ and $\mu = \frac{1}{65\,\mathrm{y}}$. Considering constant population sizes for humans ($N(t) = S(t) + I(t) + R(t)$) and for mosquitos ($M(t) = U(t) + V(t)$), the ODE system can be symplified to a three dimensional SRV system

$$\frac{d}{dt}S = \mu(N-S) - \frac{\beta}{M}SV \quad , \quad \frac{d}{dt}R = \gamma(N-S-R) - \mu R \quad , \quad \frac{d}{dt}V = \frac{\vartheta}{N}(M-V)(N-S-R) - \nu V \quad . \quad (12)$$

Once again, the dynamic of the vector is faster than the human one, so it is necessary to separate the time scales and change the parameters considering $\nu = \frac{\bar{\nu}}{\varepsilon}$ and $\gamma = \frac{\bar{\gamma}}{\varepsilon}$, and changing as well the connected parameters $\vartheta$ and $\beta$. A trajectory is plotted in state space projections in figure 2. The stationary states of the system are given by

$$V_1^* = 0 \quad \text{or} \quad V_2^* = \frac{\varepsilon\mu\left(\bar{\beta}\bar{\vartheta} - \bar{\nu}(\bar{\gamma} + \varepsilon\mu)\right)}{\bar{\beta}\left(\bar{\nu}(\varepsilon\mu + \bar{\gamma}) + \varepsilon\mu\bar{\vartheta}\right)} M \quad (13)$$

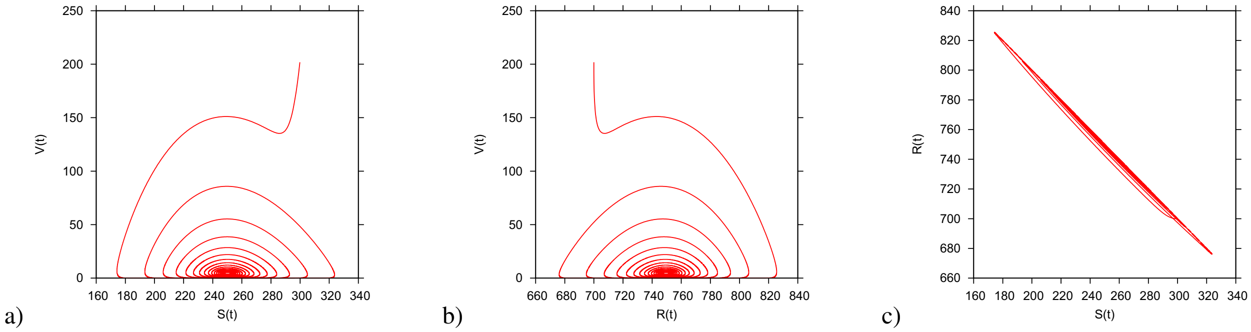

**FIGURE 2.** Projection of the simulation of the SRV model, in the three planes : a) In the plane (SV) it is possible to see that the model goes initially along the V direction before spiralling into the stationary state. b) The same behaviour is observed in the (RV) plane. c) The projection of on the (SR) plane shows that the spiralling region occur in a declinated plane.

for the extinction respectively the endemic fixed point, and the gereral formulas for the stationary states of other variables as functions of $V^*$ are $R^* = \frac{\bar{\nu}\bar{\gamma}}{\varepsilon\mu\bar{\vartheta}(M-V^*)} N V^*$ and $S^* = N\left(1 - \frac{\bar{\nu}\bar{\gamma}}{\varepsilon\mu\bar{\vartheta}(M-V^*)} V^*\left(1+\frac{\varepsilon\mu}{\bar{\gamma}}\right)\right)$. In order to verify the stability of the system we found the Jacobian matrix for the model

$$A = \begin{pmatrix} -\varepsilon\mu - \frac{\beta}{M}V^* & 0 & -\frac{\beta}{M}S^* \\ -\gamma & -(\gamma+\varepsilon\mu) & 0 \\ -\frac{\vartheta}{N}(M-V^*) & -\frac{\vartheta}{N}(M-V^*) & -\frac{\vartheta}{N}(M-S^*-R^*)-\nu \end{pmatrix} =: \begin{pmatrix} a_{11} & 0 & a_{13} \\ -\gamma & -(\gamma+\varepsilon\mu) & 0 \\ a_{31} & a_{32} & a_{33} \end{pmatrix} \quad . \tag{14}$$

The plots of figure 2 suggest that the system has two complex conjugate eigenvalues $\lambda_{1/2} = a \pm ib$ and another one which is real $\lambda_3 = c$. As the plot shows a fast approach to an invariant manifold, and then in this manifold a slow spiraling into the fixed point it is to be expected that $a \approx 0$ and $c << 0$, and the numerical simulation show that ($\lambda_{1/2} = a \pm ib = -0.029909 \pm i \cdot 0.99478$ and $\lambda_3 = c = -88.68$). In the same way, we expect two complex eigenvectors ($\underline{u}_{1/2} = \underline{v} \pm i \cdot \underline{w}$) and the other one real ($\underline{u}$). The vectors needed to calculate the eigenvectors of the model are given by

$$\underline{u} = \frac{1}{k_u}\begin{pmatrix} 1 \\ \frac{\gamma}{c-(\gamma+\mu)} \\ \frac{c-a_{11}}{a_{12}} \end{pmatrix} \approx \begin{pmatrix} 0 \\ 0 \\ 1 \end{pmatrix}, \; \underline{v} = \frac{1}{k_v}\begin{pmatrix} 1 \\ \frac{a-a_{11}-b}{a_{12}} \\ \frac{\gamma(a-(\gamma+\mu)+\gamma b)}{(a-(\gamma+\mu))b} \end{pmatrix} = \begin{pmatrix} 0.68 \\ -0.69 \\ 0.25 \end{pmatrix}, \; \underline{w} = \frac{1}{k_w}\begin{pmatrix} 1 \\ \frac{a-a_{11}+b}{a_{12}} \\ \frac{\gamma(a-(\gamma+\mu)-\gamma b)}{(a-(\gamma+\mu))b} \end{pmatrix} = \begin{pmatrix} 0.69 \\ -0.67 \\ -0.27 \end{pmatrix}$$

The real eigenvector indicates that the model goes in V direction. But the complex eigenvectors with real vectors indicate a very small angle between strongly contracting direction and the invariant manifold. This seems to reflect the close correlation between infected mosquito dynamics and human disease dynamics. Still a slaving of the fast mosquito dynamics via the slow human dynamics is observed due to the huge difference between real eigenvalue with $c = -88.68$ and the near zero real part of the other two eigenvalues $a = -0.029909$, leaving the mosquito dynamics to matter little in the approach towards the endemic fixed point.

## ACKNOWLEDGMENTS

This work has been supported by the European Union under FP7 in the projects EPIWORK and DENFREE and by FCT, Portugal, in various ways, especially via the project PTDC/MAT/115168/2009.
